# A new measure to assess pain in people with haemophilia: The Multidimensional Haemophilia Pain Questionnaire (MHPQ)

**DOI:** 10.1371/journal.pone.0207939

**Published:** 2018-11-28

**Authors:** Ana Cristina Paredes, Patrício Costa, Armando Almeida, Patrícia R. Pinto

**Affiliations:** 1 Life and Health Sciences Research Institute (ICVS), School of Medicine, University of Minho, Braga, Portugal; 2 ICVS / 3B’s–PT Government Associate Laboratory, Braga / Guimarães, Portugal; 3 Faculty of Psychology and Education Sciences, University of Porto, Porto, Portugal; Florida State University, UNITED STATES

## Abstract

People with haemophilia (PWH) experience acute pain during joint bleeds and might develop chronic pain due to joint degeneration. However, there is a lack of standardized measures to comprehensively assess pain in PWH. This study aimed to develop a multidimensional questionnaire for haemophilia-related pain, the Multidimensional Haemophilia Pain Questionnaire (MHPQ), and to present initial validation data among adults.The questionnaire distinguishes between acute/chronic pain and queries about pain locations, duration, frequency, triggering factors, intensity, interference, strategies, specialists for pain management and satisfaction with treatment. An initial version was tested with 16 patients to ensure item comprehensibility and face validity. The final version was answered by 104 adults, with 82 (78.8%) reporting haemophilia-related pain in the previous year (mean age = 43.17; SD = 13.00). The non-response analysis revealed good item acceptability. Exploratory and confirmatory factor analysis (EFA/CFA), reliability (internal consistency, test-retest, inter-item and item-total correlations) and convergent validity were analysed for the intensity and interference dimensions of the questionnaire. A combined EFA with these two constructs supported a 2-factor structure distinguishing intensity (α = 0.88) from interference items (α = 0.91). CFA was tested for the interference dimension, demonstrating suitability for this sample. Item-total correlations were >0.30 on both dimensions and most inter-item correlations were <0.70. Test-retest reliability (n = 42) was good for intensity (r = 0.88) and interference (r = 0.73), and convergent validity was confirmed for most hypotheses (r>0.30).This questionnaire is a comprehensible tool, achieving a thorough assessment of relevant pain dimensions. The MHPQ can help guide treatment recommendations by highlighting relevant topics and contributing to more effective, integrated treatments.

## Introduction

Haemophilia is an X-linked rare genetic bleeding disorder, triggered by a deficiency in coagulation factor VIII (haemophilia A) or IX (haemophilia B), and associated with a pattern of spontaneous bleeding that is the hallmark of the disease [1,2]. Haemophilia severity is defined according to coagulation factor concentration in the blood (mild: 5–40% of normal factor level; moderate: 1–5% of normal; severe: <1% of normal), which usually determines expected bleeding rate [3].

Pain is a central issue in the lives of people with haemophilia (PWH). Spontaneous joint bleeds (haemarthrosis) cause the accumulation of intra-articular blood, resulting in swelling, impaired mobility and severe acute pain [2,4]. In turn, repeated haemarthrosis progressively contribute to irreversible joint degeneration and later development of chronic haemophilic arthropathy, characterized by joint deformity, disability and chronic pain [5–7]. Therefore, pain in haemophilia can be either acute (haemarthrosis) or chronic (haemophilic arthropathy), or occur concurrently, thereby posing unique challenges to pain assessment and management. Despite its pervasiveness, pain is yet suboptimally treated among PWH, underlining the need to address this concern within the haemophilia comprehensive care setting [8,9].

Though it is consensual that a thorough pain assessment is the basis for optimal pain management, the lack of specific and validated pain tools for haemophilia is also acknowledged, in spite of the abundance of disease-specific questionnaires for other painful conditions [10–12]. This emphasizes the need to develop an assessment tool that accounts for the idiosyncrasies of pain in haemophilia.

Haemophilia-related pain has been assessed using distinct measures, from unidimensional Visual or Numerical Rating Scales [13,14], to multidimensional pain questionnaires like the McGill Pain Questionnaire [14,15] or the Brief Pain Inventory [8,16,17]. Yet, these multidimensional measures might not be sensible to haemophilia-related pain idiosyncrasies, such as the co-occurrence of acute and chronic pain and the potential report of multiple pain locations. Other questionnaires containing pain subscales have also been used, such as general [16,18,19] and disease-specific [20,21] quality-of-life questionnaires, or specific joint assessment measures, like the Gilbert Score [22] and the Haemophilia Joint Health Score [23]. Previous works have relied on unstandardized questions developed for specific investigation aims [13,24,25] and there is also a former work reporting the development of a haemophilia pain questionnaire [26]. However, and to our knowledge, these measures have not been further validated or systematically implemented in the haemophilia field.

Additionally, the few studies addressing pain assessment among children with haemophilia [27] usually rely on the Wong-Baker FACES Scale [28,29]. Since this is a unidimensional pain intensity scale, it disregards information such as pain duration, frequency, triggering factors or interference.

Meanwhile, strong calls to action have been made to improve pain assessment and management in the haemophilia field, with several gaps being pointed out by research and journal editorials [30–32]. Of greater emphasis has been the need to clearly distinguish acute from chronic pain, to develop and validate pain assessment instruments appropriate for PWH of all ages, and to treat patients in multidisciplinary comprehensive teams that include pain specialists [9,31,33]. This would ultimately contribute to uncover specific intervention needs and targets, thereby improving haemophilia-related pain management and patients’ care [13,30,31].

This study aimed to develop a multidimensional questionnaire suitable for haemophilia-related pain across all ages, and to present initial validation data among adults.

## Methods

### Questionnaire development

The Multidimensional Haemophilia Pain Questionnaire (MHPQ) was developed within a biopsychosocial framework and following IMMPACT (Initiative on Methods, Measurement, and Pain Assessment in Clinical Trials) recommendations for pain measures [34,35]. Furthermore, it aimed to answer the calls and circumvent the gaps concerning haemophilia pain assessment, by considering the nature of haemophilia-related pain and the distinct pain dimensions that are recommended to be considered when assessing pain [10,35].

For the development of this measure, item generation resulted from careful consideration of target population and study aims, and was grounded on an extensive review of published literature and existing pain questionnaires [26,36,37], along with meetings with medical experts in haemophilia care, and our team’s experience in assessing patients with pain [38–42]. At the initial stage, a set of items was either retrieved from other well-validated pain questionnaires or originally developed, aiming to cover well-established important pain dimensions, such as: location, duration, frequency, triggering factors, intensity, interference, coping strategies and satisfaction with pain treatment [10,35]. The resulting item pool was later piloted with a group of 16 patients for comments on comprehensibility and relevance, and to collect further item suggestions.

In this line, developing a questionnaire suitable for all ages was also a priority, yet some age-related adaptations had to be considered. For instance, age-appropriate examples were provided and adapted for each version, to facilitate item understanding. In what concerns content and format, the adult (≥18 years) and the children/teenagers (10–17 years) versions were equal. In turn, to assess children from 0 to 9 years old, we propose a proxy version that should be completed by a parent or caregiver. This proxy version differs from the others by not assessing neither pain intensity nor degree of relief from pain strategies, and the interference subscale is rated on a 5-point Likert scale. Although we are aware of possible constraints in assessing pain by proxy, we also believe that this is relevant information for healthcare providers and researchers.

#### Pilot study

The questionnaire underwent a cognitive debriefing process with 16 patients with haemophilia who were interviewed individually. This process aimed to account for face validity of the questionnaire, ensuring that the instructions, item wording and format were clearly understood, and that the proposed items are relevant for PWH of all age ranges. Interviews were conducted until data saturation was reached, and were performed with six adults, six children/teenagers (10 to 17 years old) and four parents of children with haemophilia (proxy version), so that all age ranges could be represented. Revisions were made to item content and structure according to patients’ and caregivers’ feedback, in order to reach a final version. Main alterations included instruction simplification and items reframing. No items were considered irrelevant or incomprehensible by the participants and no additional questions were suggested.

#### Final version

The final version of the MHPQ assesses haemophilia-related pain and was reached after both clinical experts and patients from pilot study have considered that the idiosyncrasies of haemophilia were fully covered by the set of questions included in the final version. It comprises four initial items aiming at the accurate distinction between acute and chronic pain, followed by nine dimensions centred on a thorough assessment of pain features. Each dimension in analysed separately and no global pain score is computed for the MHPQ.

The following dimensions are included in the final version of the questionnaire:

*-* Acute vs. chronic pain: The first 4 items aim to evaluate the presence of chronic pain, according to the guidelines of the European Haemophilia Therapy Standardization Board (EHTSB). These 4 items were designed to match the specific requirements for chronicity suggested by the EHTSB. Jointly, they regard the association between reported pain and the pathophysiology of haemophilia, the three-month cut-off point and a frequency higher than once a week [10]. If no pain is reported during the previous year, the remaining questionnaire is not answered. For patients reporting pain in the previous year, subsequent questions are answered reporting to that period, since in the case of haemophilia it has been suggested that pain should be assessed for longer periods of time [27].

*-* Pain locations: query about all haemophilia-related pain locations, allowing for the inference of a “number of painful locations" measure. Furthermore, this section also requires the selection of the most painful location and of the location in which pain caused the greatest impact in the previous year. The remaining questions on duration, frequency, triggers, intensity and interference should be answered according to the pain that caused the greatest impact.

*-* Duration: asks about how long ago the pain with greatest impact started, with an open-ended question.

*-* Frequency (and temporal pattern): enquires about how often the pain occurs (*e*.*g*. daily, weekly, only during bleeds), the time of day when it hurts most and when was the last time pain was experienced.

*-* Triggering factors: implies the selection, from a list, of perceived triggers associated with pain onset, such as: bleeds, climbing stairs, “wrong” movements or weather changes.

*-* Intensity: evaluated according to six specific conditions (bleeding episodes; physical efforts and/or movements; using stairs; after resting or staying still; during rest, sitting or lying down; and accidental or “wrong” movements), corresponding to each of the triggering factors previously presented, through a 0–10 Numerical Rating Scale (NRS) (0 = no pain; 10 = worst imaginable pain). This dimension only figures on the self-report versions. An average total score can be computed to obtain an intensity global score.

*-* Interference: assessed using the interference dimension items integrally retrieved from the Brief Pain Inventory (BPI) [37], which is a widely used measure for pain evaluation that has been previously translated, adapted and validated for European Portuguese [43]. Participants rate the interference of haemophilia-related pain in seven domains: general activity, mood, walking ability, normal work, relations with other people, sleep and enjoyment of life. In self-report questionnaires, the items are rated according to the original 0–10 NRS (0 = no interference; 10 = completely interferes) and, in proxy versions, the same items are answered on a 5-point qualitative scale ranging from 1 –“does not interfere”, to 5 –“completely interferes”. An additional “does not apply” option is also present if that item is not applicable to the child (*e*.*g*. walking/crawling ability). Also, item content is adapted to age-specific activities when appropriate (*e*.*g*. work vs. school work). An average total score can be reported as a global interference score, by computing the average of responses.

- Strategies for pain management: presents a list of several pharmacological and non-pharmacological strategies from where participants are required to select those they usually do or ever did. Following each elected strategy, patients indicate the degree of perceived relief, on a 0–100% scale. The strategies presented are based on general recommendations for the management of acute and chronic pain related to haemophilia [*e*.*g*. rest, ice, compression and elevation (RICE)], as well as on other common options for pain control (*e*.*g*. analgesics and ointments), including the use of alternative therapies (*e*.*g*. reiki) and other pain coping strategies (*e*.*g*. drinking alcohol, distracting, seeking company/support from family and friends).

- Pain management specialists: asks which health care professionals, or other specialists, people have consulted, or would like to consult, to assist in pain management. A list of 11 specialists is presented, including haemophilia doctors, anaesthesiologists, psychologists, physical therapists and professionals of alternative therapies (*e*.*g*. acupuncture, meditation and reiki).

*-* Satisfaction with pain treatment: assesses global satisfaction with pain treatment through a single question, answered on a 5-point scale ranging from 1 –“very dissatisfied” to 5 –“very satisfied”.

### Participants and procedures

This was an observational and longitudinal prospective study conducted among PWH who participated in the first haemophilia survey implemented in Portugal [44], which included male participants with haemophilia A or B of all ages. Exclusion criteria were inability to read and write or to consent voluntary participation.

The surveys were sent by mail to 500 PWH and returned by 146 participants (29.2% return rate). Answers to the survey were received from October 2016 through May 2017 and those participants authorizing subsequent contacts from the research team were reached after the first collaboration and invited to fill in the pain questionnaire for a second time, three months after the initial assessment.

Though pain information was collected from PWH of all ages, this report will focus exclusively on data concerning adults. Due to low sample size on younger ages, stemming from the fact that this is a rare disease with a prevalence of approximately 700 cases in Portugal [45], the validation study for these groups was not performed.

From the 106 adults participating in the first assessment time, two were excluded due to a large amount of missing data, and 22 did not report pain due to haemophilia in the previous year. Therefore, they were not included in the validation process of this pain questionnaire, leaving 82 adult men in the sample under analysis.

Approval for this study was obtained by the Ethical Committee at University of Minho and by the Portuguese Data Protection Agency, and it is registered at clinicaltrials.gov (NCT02870114). Informed consent was obtained from the participants or legal guardians. More detailed information concerning survey procedure and patient recruitment can be found elsewhere [44].

### Measurements, assessments and instruments

- Sociodemographic and Clinical questionnaire: collects information regarding sociodemographic (*e*.*g*. age, education, professional status) and clinical (*e*.*g*. disease type and severity, bleeding episodes, affected joints) characteristics.

- Patient-Reported Outcomes Measurement Information System (PROMIS)–Anxiety and Depression (short forms) [46]: each measure has 4 items that assess symptoms of anxiety (α = 0.85) and depression (α = 0.92) such as fear (anxiety) or hopelessness (depression). Scores range from 4 to 20, with higher scores indicating more severe symptoms.

- A36 Hemofilia-QoL [20]: assesses health-related quality-of-life in PWH through 36 items, divided in nine subscales. For the purpose of this validation work, only the global scale (α = 0.96) and the following subscales will be considered: daily activities (α = 0.94), joints (α = 0.82), pain (α = 0.80), emotional functioning (α = 0.84), mental health (α = 0.82) and relationships and social activity (α = 0.91). The items are scored according to a 5-point Likert scale, with higher values translating better quality-of-life.

- Haemophilia Activities List (HAL) [15]: evaluates patients’ self-reported functional ability, namely the difficulty in performing activities due to haemophilia. In this study, only the following subscales were included in the analyses: lying/siting/kneeling/standing (LSKS) (α = 0.94), function of the legs (α = 0.96), function of the arms (α = 0.92), household tasks (α = 0.85) and leisure activities and sports (α = 0.80). Scores for the subscales and the global scale (α = 0.97) range from 0 (worst functional status) to 100 (best functional status).

- Illness Perception Questionnaire-Revised (IPQ-R) [47]: assesses patients’ beliefs and perceptions about haemophilia, according to seven subscales. For the purposes of this study, only the consequences (α = 0.69) and emotional representation (α = 0.83) subscales were included in the analyses. Each item is answered on a 5-point scale and scores for each dimension range between 3 and 15, with higher values translating more threatening illness perceptions.

### Statistical analysis

Data were analysed using the IBM SPSS Statistics and the IBM SPSS AMOS versions 24 (Chicago, IL, USA).

Data were presented as absolute and relative frequencies (n; %) for categorical data, and both as mean±standard deviation and median (min-max) for continuous items. An independent t-test analysis was computed to test for differences between respondents and non-respondents to the follow-up assessment. The statistical level of significance was established at p<0.05.

#### Item statistics

Item-level analyses were conducted to test the psychometric properties of items. Item normality was assessed using skewness and kurtosis, with values between -1.5 and 1.5 being accepted as normally distributed [48]. The number of missing values (response frequency) was examined to assess if the items were well accepted and descriptive statistics were computed (mean, median, standard deviation, skewness and kurtosis). Missing values in the interference dimensions (n = 4) were replaced by the participants median value in order to perform the confirmatory factory analysis (CFA).

#### Exploratory and confirmatory factor analysis (EFA/CFA)

The 13 items assessing pain intensity and pain interference were firstly included in EFA, in order to determine the number of factors that resulted from this analysis. EFA was performed with principal axis factoring with an oblique rotation method (Oblimin), and considering as an adequate sample size a subject-to-variable ratio of 5:1 [49].

Specifically for interference items, and considering that the BPI interference dimension has been previously validated and showed good psychometric properties among Portuguese patients [43], CFA was adopted to assess its suitability for this sample. The model was evaluated considering the following goodness-of-fit indices and thresholds: chi-square (χ^2^;p>0.05), χ^2^/degrees of freedom ratio (≤3), Normed Fit Index (NFI≥0.90), Tucker–Lewis Index (TLI≥0.90), Comparative Fit Index (CFI≥0.90), Root Mean Squared Error of Approximation (RMSEA<0.08) and Standardized Root Mean Squared Residual (SRMR≤0.08) [50,51]. Covariance between errors was added according to modification indices (MI), when MI>11 [χ^2^0.999; (1) = 10.83].

#### Reliability

Internal consistency of responses was assessed using Cronbach’s alpha (values over 0.70 are considered indicators of sufficient item homogeneity) [52]. Item-total and inter-item correlations were computed, considering cut-off values over 0.30 and under 0.70, respectively [53,54].

Three months after the first participation, a total of 43 adults answered the questionnaire to assess test-retest reliability. This was analysed by computing Pearson correlation tests, based on a 0.70 threshold [54].

#### Convergent validity

To examine the convergent validity of the MHPQ, Pearson correlation coefficients and point-biserial correlation coefficients were computed between those MHPQ dimensions that have a score (number of painful locations, intensity and interference), the validating measures previously described and clinical outcomes. Hypotheses concerning expected relationships were developed *a priori* and convergent validity was assumed if a medium or strong (r≥0.30) significant correlation was found [55].

We hypothesised that the number of painful locations, pain intensity and pain interference would be: a) positively correlated with the clinical outcomes assessed at baseline (number of days hospitalised, bleeding episodes, affected joints and opioid prescription); b) positively correlated with PROMIS anxiety and depression scores, with the emotional representation and consequences subscales of the IPQ-R; and c) negatively correlated with functionality (HAL global score, LSKS, function of the legs, function of the arms, household tasks, leisure activities and sports) and with quality-of-life (A36 Hemofilia-QoL global score, daily activities, joints, pain, emotional functioning, mental health and relationships and social activity). Concerning the clinical outcomes assessed later (3 month follow-up), it was hypothesised that the MHPQ dimensions would be: d) positively correlated with the number of bleeding episodes, number of affected joints and weekly pain frequency.

## Results

### Participants

One-hundred and four adults with haemophilia completed the first haemophilia national survey in Portugal, of which 82 (78.8%) reported to have had pain due to haemophilia in the previous year. Furthermore, 65 (62.5%) participants indicated that their pain lasted over three months and 43 (41.3%) stated that pain occurred more than once a week (Table 1). For the purposes of this validation study, only those who reported pain in the previous year (N = 82) were considered.

**Table 1:** Prevalence of pain due to haemophilia among study participants (N = 104).

Lifetime pain	93 (89.4%)
In the previous year	82 (78.8%)
Lasting over three months	65 (62.5%)
More than once a week	43 (41.3%)

Table 2 reveals that the mean age of participants was 43.17 years old (SD = 13.00) and most of them had completed high school education or higher (61; 74.4%). Concerning occupation status, 49 (60.5%) participants reported a full or part-time occupation and, of those who were unemployed, retired or on medical leave, 20 (62.5%) were in that situation due to haemophilia. Regarding clinical characteristics, the majority of participants had haemophilia A (73; 89%) and severe haemophilia was reported by 54 (65.9%) participants. Full socio-demographic and clinical characteristics of the sample are summarized on Table 2.

**Table 2:** Sociodemographic and clinical characteristics of study participants (Baseline, N = 82^a^).

**Age**	43.17±13.00 44 (18–74)
**Education:** completed level, n (%)	
Primary school (1^st^-4^th^ grade)	5 (6.1)
Middle school (5^th^-9^th^ grade)	16 (19.6)
High school (10^th^-12^th^ grade)	33 (40.2)
College/Postgraduate degree	28 (34.2)
**Marital status:** married, n (%) [80]	46 (57.5)
**Professional status,** n (%) [81]	
Full or part-time occupation	49 (60.5)
Unemployed	8 (9.9)
Retired	22 (27.2)
Medical leave	2 (2.5)
If unemployed/retired/ medical leave (n = 32)	
Due to haemophilia, n (%)	20 (62.5)
**Type of haemophilia,** n (%)	
Haemophilia A	73 (89)
Haemophilia B	9 (11)
**Haemophilia severity,** n (%)	
Mild	7 (8.5)
Moderate	21 (25.6)
Severe	54 (65.9)
**Inhibitors:** Yes, n (%) [78]	14 (17.9)
**Prophylaxis treatment:** Yes, n (%)	31 (37.8)
**Hospitalisation due to haemophilia in previous year**: Yes, n (%)	10 (12.2)
Number of days	13.50±12.71 7.5 (4–39)
**Bleeding episodes in previous year:** Yes, n (%) [72]	65 (90.3)
Number of bleeding episodes	15.74±17.33 10 (1–84)
**Joint deterioration:** Yes, n (%)	80 (97.6)
Number of affected joints	4.40±2.55 4 (1–10)
**Opioid prescription:** Yes, n (%) [76]	15 (19.7)

Note: Categorical variables are presented as n (%). Continuous variables are presented as mean ± standard deviation and median (min-max).

^a^ Unless otherwise specified in square brackets.

### Pain characteristics of adult PWH

Tables 3 to 7 provide a description of item content and show the results obtained in the present sample concerning pain locations, duration, frequency and triggering factors (Table 3); pain intensity and pain interference (Table 4); pain management strategies (Table 5) and specialists (Table 6); and satisfaction with pain treatment (Table 7).

**Table 3:** Pain characteristics of study participants (reporting to the previous year, Baseline, N = 82^a^).

**Pain locations**^b^	Left, n (%)	Right, n (%)	Laterality not specified, n (%)
Ankle	53 (64.6)	47 (57.3)	
Elbow	35 (42.7)	46 (56.1)	
Knee	36 (43.9)	37 (45.1)	
Shoulder	23 (28.0)	26 (31.7)	
Hip	15 (18.3)	19 (23.2)	
Wrist	11 (13.4)	17 (20.7)	
Muscles			4 (4.9)
Other locations^c^			7 (8.5)
**Most painful locations**^b^			
Ankle	20 (24.4)	21 (25.6)	
Knee	19 (23.2)	19 (23.2)	
Elbow	5 (6.1)	10 (12.2)	
Shoulder	3 (3.7)	4 (4.9)	
Hip	3 (3.7)	4 (4.9)	
Wrist	1 (1.2)	2 (2.4)	
Muscles			1 (1.2)
Other locations^c^			1 (1.2)
**Pain location with more impact**			
Ankle	15 (18.3)	16 (19.5)	
Knee	15 (18.3)	15 (18.3)	
Elbow	2 (2.4)	6 (7.3)	
Hip	1 (1.2)	4 (4.9)	
Shoulder	2 (2.4)	3 (3.7)	
Muscles			1 (1.2)
Other locations^c^			2 (2.4)
**Number of pain locations**	5.23±3.95 4 (1–16)		
**Pain duration** (months) [63]	137.70 ± 136.46 96 (1–612)		
**Pain frequency**^b^	n (%)		
During physical efforts and/or movement	34 (41.5)		
After getting hurt or during bleeds	20 (24.4)		
Weekly, but not daily	17 (20.7)		
Daily, but not constant	15 (18.3)		
Always present, continuous, constant	12 (14.6)		
**Last time in pain** [81]	n (%)		
Today	27 (33.3)		
Last week	20 (24.7)		
Last month	15 (18.5)		
1–6 months	13 (15.9)		
6–12 months	6 (7.4)		
**Pain temporal pattern**^b^ [80]	n (%)		
Depends	48 (60)		
Night	17 (21.3)		
Morning	15 (18.8)		
End of the day	11 (13.8)		
Afternoon	5 (6.3)		
**Pain triggering factors**^b^	n (%)		
During physical efforts and/or movement	61 (74.4)		
Accidental or “wrong” movements	45 (54.9)		
Bleeding episode	43 (52.4)		
After resting or staying still	41 (50)		
Weather changes	34 (41.5)		
Using stairs	33 (40.2)		
During rest, sitting or lying down	18 (22)		
Always present, constant	10 (12.2)		

Note: Categorical variables are presented as n (%). Continuous variables are presented as mean ± standard deviation and median (min-max).

^a^ Unless otherwise specified in square brackets

^b^ More than one response option was possible

^c^ Other pain locations include the back, groin, abdomen and mouth.

**Table 4:** Descriptive statistics and Cronbach’s alpha of the pain intensity and interference items (Baseline, N = 82).

**Pain intensity (0–10 NRS)**	N	M±SD	Md	min-max	Sk	K	α*
Total subscale	76	4.34±2.06	4.50	0.67–9.5	0.127	-0.462	0.879
1. Bleeding episodes	81	5.67±2.09	6.00	0–10	-0.384	-0.143	0.890
2. During physical efforts and/or movement	81	5.19±2.37	6.00	0–10	-0.392	-0.220	0.848
3. Using stairs	80	4.21±2.95	4.00	0–10	0.111	-0.980	0.841
4. After resting or staying still	79	3.65±2.73	4.00	0–10	0.201	-0.847	0.851
5. During rest, sitting or lying down	79	2.20±2.48	1.00	0–9	0.778	-0.546	0.864
6. Accidental or “wrong” movements	81	5.35±2.97	6.00	0–10	-0.275	-0.925	0.844
**Pain interference (0–10 NRS)**	N	M±SD	Md	min-max	Sk	K	α*
Total subscale	79	4.14±2.34	4.43	0.14–9.9	0.056	-0.864	0.906
1. General activity	82	4.67±2.69	5.00	0–10	0.071	-0.765	0.887
2. Mood	82	4.13±2.80	4.00	0–10	0.477	-0.391	0.898
3. Walking ability	80	5.65±3.23	6.00	0–10	-0.249	-1.086	0.893
4. Normal work	80	4.99±2.70	5.00	0–10	0.101	-0.820	0.885
5. Relations with other people	82	2.93±2.79	2.00	0–10	0.680	-0.552	0.894
6. Sleep	82	3.29±3.13	2.00	0–10	0.611	-0.819	0.896
7. Enjoyment of life	82	3.71±3.21	4.00	0–10	0.348	-1.069	0.891

*Cronbach’s alpha reported for total scale and if item deleted

**Table 5:** Strategies for pain management and perception of relief (Baseline, N = 82).

Strategies for pain control	Yes, n (%)	Relief (0–100%)				
		M±SD	Md	min-max	Sk	K
Ice	71 (86.6)	43.42±24.47	50.00	3–100	0.172	-0.392
Rest	68 (82.9)	49.00±21.58	50.00	0–100	-0.291	-0.222
Clotting factor replacement	66 (80.5)	77.81±23.09	80.00	0–100	-1.693	2.711
Pain medication	62 (75.6)	59.33±23.67	60.00	5–100	-0.441	-0.378
Elevation	39 (47.6)	33.05±20.71	30.00	0–80	0.332	-0.796
Compression	22 (26.8)	25.00±19.00	20.00	0–70	0.998	0.545
Distracting	21 (25.6)	35.68±20.80	40.00	3–75	-0.111	-0.785
Relaxing techniques	19 (23.3)	43.89±23.61	50.00	5–80	-0.451	-1.056
Search for support/company	12 (14.6)	48.33±30.33	45.00	5–100	0.214	-0.652
Complementary therapies^a^	12 (14.6)	36.04±30.70	25.00	0–80	0.368	-1.645
Substance use^b^	10 (12.2)	30.10±23.04	30.00	0–75	0.719	0.261
Heat	8 (9.8)	42.86±18.00	50.00	10–60	-1.074	0.701
Praying	8 (9.8)	21.13±34.08	10.00	0–99	2.149	4.642

Note

^a^Includes acupuncture, therapeutic massage, reiki/meditation and natural products/homeopathy/naturopathy

^b^ Includes alcohol, tobacco and recreational drugs.

**Table 6:** Pain management specialists who participants consulted or wished to consult to deal with haemophilia-related pain (Baseline, N = 82).

Pain Specialists	Have Consulted, n (%)	Wish to consult, n (%)
Haemophilia doctor	71 (86.6)	2 (2.4)
Orthopaedist	63 (76.8)	1 (1.2)
Family doctor	40 (48.8)	0
Physical therapy/Physiotherapist	36 (43.9)	6 (7.3)
Physiatrist	28 (34.1)	5 (6.1)
Psychologist	9 (11)	2 (2.4)
Anaesthesiologist	7 (8.5)	1 (1.2)
Reiki specialist	5 (6.1)	6 (7.3)
Meditation specialist	5 (6.1)	3 (3.7)
Acupuncture specialist	3 (3.7)	9 (11)
Psychiatrist	3 (3.7)	0
Other specialists	3 (3.7)	1 (1.2)
Have not consulted any pain specialist, to help deal with haemophilia-related pain: 3 (3.7)		

**Table 7:** Satisfaction with current pain treatment by health care professionals (Baseline, N = 82).

	n (%)
Very satisfied	8 (10)
Satisfied	31 (38.8)
Neither satisfied nor dissatisfied	29 (36.3)
Dissatisfied	7 (8.8)
Very dissatisfied	5 (6.3)

### Evaluation of measurement properties

#### Item statistics

Item acceptability and face validity were preliminarily assessed in the pilot test phase of questionnaire development, considering patients’ feedback on comprehensibility and relevance.

Given the discrete nature of the variables assessed, item distribution was expected to demonstrate some degree of non-normality. For all the 13 items assessing intensity and interference, skewness varied from -0.392 (intensity-item 2) to 0.778 (intensity-item 5) and kurtosis ranged from -1.086 (interference-item 3) to -0.143 (intensity-item 1) (Table 4). These results reveal acceptable values for both skewness and kurtosis (within the range of +/- 1.5), showing no severe violation of normality.

Concerning pain management strategies, the degree of relief provided by most strategies was normally distributed, except for “clotting factor replacement” (Sk = -1.693; K = 2.711) and “praying” (Sk = 2.149; K = 4.642) (Table 5).

The percentage of missing responses to all items was analysed as an indicator of their relevance. Missing responses were found on three items focusing on pain characteristics: pain duration (19; 23.2%), last time in pain (1; 1.2%) and pain temporal pattern (2; 2.4%). Missing values in the intensity and interference subscales were found for six (7.3%) and three participants (3.7%), respectively.

#### Exploratory and confirmatory factor analysis (EFA/CFA)

Findings from EFA including all intensity and interference items are described in Table 8. The results supported a 2-factor structure distinguishing the intensity items from the interference items, showing that the items are assessing two different constructs, as expected.

**Table 8:** Obliquely rotated factor loadings of principal axis factoring for the 13 items assessing pain intensity and interference.

	Factor 1	Factor 2
Bleeding episodes	-0.028	**0.513**
During physical efforts and/or movement	-0.020	**0.821**
Using stairs	0.096	**0.784**
After resting or staying still	-0.027	**0.784**
During rest, sitting or lying down	0.027	**0.670**
Accidental or “wrong” movements	0.125	**0.738**
General activity	**0.686**	0.183
Mood	**0.775**	-0.080
Walking ability	**0.723**	0.063
Normal work	**0.744**	0.122
Relations with other people	**0.902**	-0.155
Sleep	**0.630**	0.161
Enjoyment of life	**0.745**	0.066
Eigenvalue	6.862	1.588
% Variance explained	49.814	9.047

Extraction method: Principal axis factoring; Rotation method: Oblimin

Note: Primary factor loadings appear in bold

**Fig 1:**
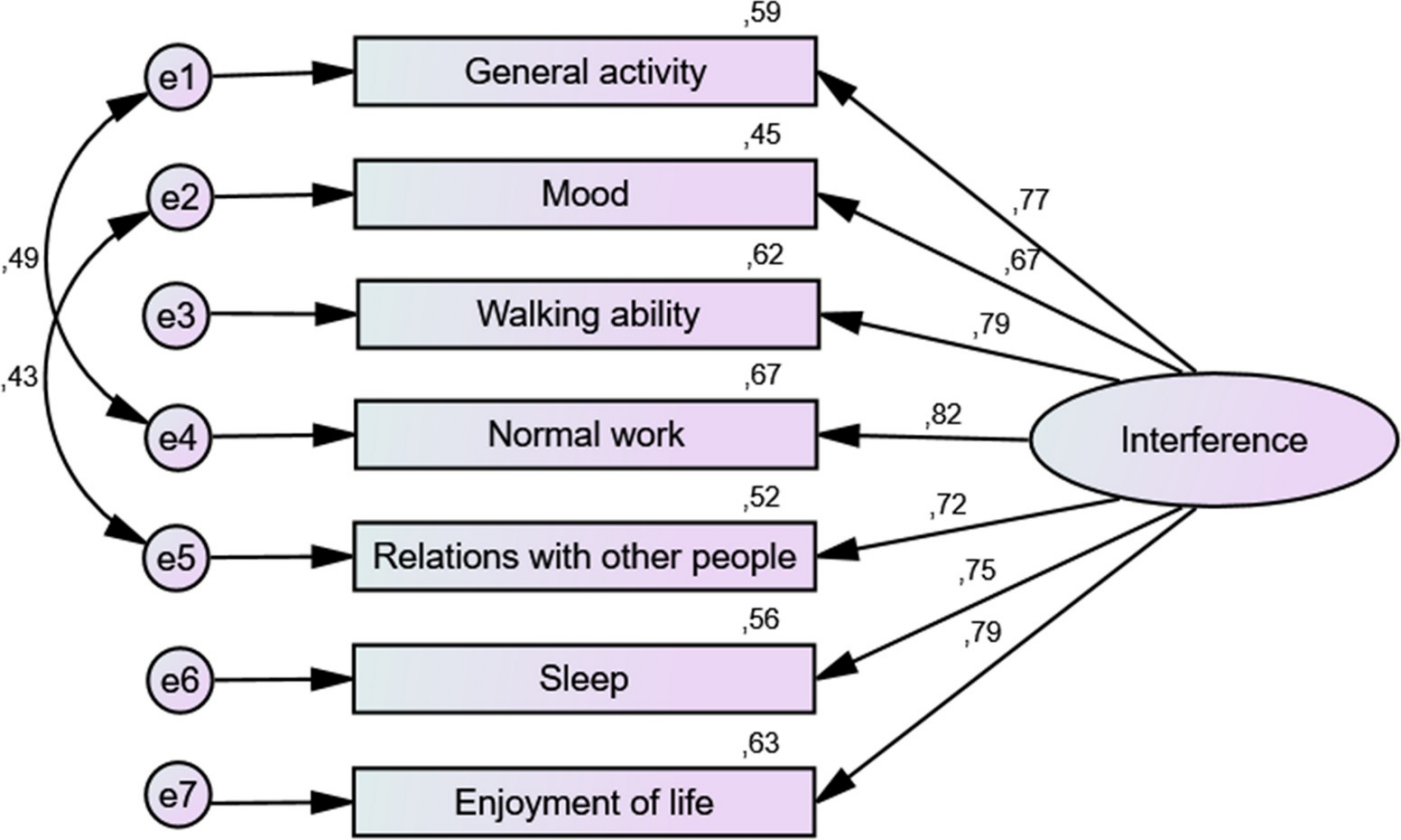
Standardized values of the confirmatory factor analysis for interference.

Two CFA models were tested for the interference dimension: Model A, with non-correlated errors, and Model B, with error terms established according to modification indices (MI). A covariance was added between two error pairs with MI>11: e1-e4 (MI = 12.35) and e2-e5 (MI = 16.34) (Fig 1). Table 9 shows goodness-of-fit measures for both models, with Model B revealing more satisfactory measures: χ^2^(12) = 15.74, p = 0.203; χ^2^/df = 1.31; NFI = 0.957; TLI = 0.981; CFI = 0.989; RMSEA = 0.062; SRMR = 0.0342.

**Table 9:** Fit indices for CFA model.

Fit indices	χ^2^	*df*	χ^2^*/df*	*p*	NFI	TLI	CFI	RMSEA	SRMR
Model A	45.701	14	3.264	<0.001	0.876	0.864	0.909	0.167	0.0606
Model B	15.741	12	1.312	0.203	0.957	0.981	0.989	0.062	0.0342

Abbreviations: χ^2^, Chi-squared; *df*, degrees of freedom; NFI, Normed Fit Index; TLI, Tucker–Lewis Index; CFI, Comparative Fit Index; RMSEA, Root Mean Squared Error of Approximation; SRMR, Standardized Root Mean Squared Residual.

#### Reliability

To assess internal consistency of the intensity and interference dimensions, Cronbach’s alpha was calculated for each subscale. Both subscales presented good reliability scores, with a Cronbach’s alpha of 0.88 for the intensity subscale and of 0.91 for the interference subscale, indicating an appropriate homogeneity of items. In both subscales, removal of any item would decrease total Cronbach’s alpha, except for “bleeding episodes” in the intensity subscale (see Table 4).

Table 10 shows that inter-item correlations for the intensity dimension were all below 0.70, indicating non-redundancy of items. Similar results were found for interference, except between the items 1–4 (general activity—normal work), 2–5 (mood—relations with other people) and 3–4 (walking ability—normal work), that had correlations above 0.70 (Table 11). Item-total correlations were greater than 0.30 on both dimensions, showing an adequate correlation of each item with its dimension and suggesting adequate scale homogeneity.

**Table 10:** Results of Pearson correlation tests for inter-item and item-total correlations of pain intensity.

	Item 1	Item 2	Item 3	Item 4	Item 5	Item 6	Global subscale
1. Bleeding episodes	1						0.586***
2. During physical efforts and/or movement	0.489***	1					0.828***
3. Using stairs	0.391***	0.693***	1				0.866***
4. After resting or staying still	0.314**	0.553***	0.688***	1			0.822***
5. During rest, sitting or lying down	0.325**	0.532***	0.543***	0.679***	1		0.755***
6. Accidental or “wrong” movements	0.480***	0.669***	0.693***	0.595***	0.535***	1	0.852***

** p≤0.01

***p≤0.001

**Table 11:** Results of Pearson correlation tests for inter-item and item-total correlations of pain interference.

	Item 1	Item 2	Item 3	Item 4	Item 5	Item 6	Item 7	Global subscale
1. General activity	1							0.832***
2. Mood	0.525***	1						0.751***
3. Walking ability	0.628***	0.459***	1					0.804***
4. Normal work	0.809***	0.527***	0.716***	1				0.849***
5. Relations with other people	0.547***	0.705***	0.545***	0.514***	1			0.784***
6. Sleep	0.612***	0.521***	0.541***	0.633***	0.540***	1		0.781***
7. Enjoyment of life	0.577***	0.589***	0.622***	0.575***	0.656***	0.593***	1	0.816***

***p≤0.001

Regarding test-retest reliability, good results were obtained both for the intensity (r = 0.878) and interference (r = 0.728) dimensions. There were no statistically significant differences between the participants who responded or did not respond to the follow-up assessment in terms of sociodemographic (age and education) and clinical (haemophilia severity, number of affected joints and number of bleeds in the previous year) characteristics (results not shown).

#### Convergent validity

Table 12 shows the results of Pearson and point-biserial correlation tests for convergent validity analysis. At least medium correlations (r≥0.30) were found between the MHPQ subscale “painful locations” and the clinical outcomes bleeding episodes (r = 0.325, p = 0.005), affected joints (r = 0.579, p<0.001) and opioid prescription (r = 0.401, p<0.001). Likewise, there were medium correlations between opioid prescription and the MHPQ subscales “pain intensity” (r = 0.309, p = 0.009) and “pain interference” (r = 0.423, p<0.001) (hypothesis a). Statistically significant (p<0.05) correlations were also found between “painful locations” and number of days hospitalised; and between “pain intensity” and “pain interference”, and number of affected joints, but only with a small correlation coefficient (r<0.30).

**Table 12:** Convergent validity between MHPQ dimensions and validating measures.

	Painful locations	Pain intensity	Pain interference
Outcomes at baseline assessment			
Number of days hospitalised^a^	0.230*	0.144	0.213
Bleeding episodes^a^	0.325**	0.024	0.117
Number of affected joints	0.579***	0.265*	0.267*
Opioid prescription	0.401***	0.309**	0.423***
PROMIS Anxiety	0.251*	0.345**	0.466***
PROMIS Depression	0.198	0.375***	0.469***
A36 Hemofilia-QoL_Global score	-0.517***	-0.560***	-0.670***
A36 Hemofilia-QoL _Daily activities	-0.483***	-0.428***	-0.578***
A36 Hemofilia-QoL _Joints	-0.515***	-0.523***	-0.502***
A36 Hemofilia-QoL _Pain	-0.519***	-0.544***	-0.459***
A36 Hemofilia-QoL _Emotional functioning	-0.337**	-0.582***	-0.583***
A36 Hemofilia-QoL _Mental health	-0.357***	-0.511***	-0.588***
A36 Hemofilia-QoL _ Relationships and social activity	-0.364***	-0.419***	-0.533***
HAL_Global score	-0.599***	-0.568***	-0.579***
HAL_Lying, Kneeling, Sitting, Standing	-0.485***	-0.504***	-0.490***
HAL_Function of the legs	-0.466***	-0.548***	-0.561***
HAL_Function of the arms	-0.586***	-0.512***	-0.458***
HAL_Household tasks	-0.626***	-0.532***	-0.526***
HAL_Leisure activities and sports	-0.447***	-0.391***	-0.493***
IPQ-R_Consequences	0.446***	0.474***	0.566***
IPQ-R_Emotional representation	0.215	0.409***	0.465***
Follow-up assessment (3 months)			
Bleeding episodes^b^	0.393**	0.245	0.231
Number of affected joints	0.585***	0.424**	0.384**
Weekly pain	0.416**	0.598***	0.464**

^a^During the previous year

^b^ During the previous 3 months

*p≤0.05

**p≤0.01

***p≤0.001

Abbreviations: PROMIS, Patient-Reported Outcomes Measurement System; HAL, Haemophilia Activities List; IPQ-R, Illness Perceptions Questionnaire Revised

Concerning psychological questionnaires, the “pain intensity” and “pain interference” MHPQ dimensions correlated positively (hypothesis b) with anxiety and depression (PROMIS), with the perception of consequences (IPQ-R) and with emotional representation underlying haemophilia (IPQ-R), ranging from r = 0.345 (p = 0.24) to r = 0.566 (p<0.001). For the “painful locations” dimension, there were positive correlations with anxiety (PROMIS) (r = 0.251, p = 0.024) and the perception of consequences (IPQ-R) (r = 0.446, p<0.001). Negative correlations (hypothesis c) were found with all functionality (HAL) and quality-of-life (A36 Hemofilia-QoL) dimensions (including the pain subscale) and global scores, ranging from r = 0.337 (p = 0.002) to r = -0.670 (p<0.001). Positive correlations were also found with bleeding episodes (only for painful locations), affected joints and weekly pain frequency (hypothesis d) assessed at follow up, ranging from r = 0.384 (p = 0.16) to r = 0.598 (p<0.001).

## Discussion

This study reports the development and initial validation of the Multidimensional Haemophilia Pain Questionnaire (MHPQ), conceived to capture the idiosyncrasies of haemophilia-related pain among PWH. It was developed under a biopsychosocial framework, following IMMPACT guidelines and haemophilia-specific recommendations, and integrating feedback from haemophilia experts and patients. It contributes to answer the calls-to-action made on the need to improve haemophilia-related pain assessment and management, bridging a recognized gap in this area and contributing to fill a long-standing need on this field [30–32]. The development process aimed to encompass and distinguish acute and chronic pain and to consider the presence of multiple pain locations, achieving a complete assessment of pain dimensions, while maintaining easy usability and avoiding patient burden [31]. Globally, the findings reveal that the MHPQ is a comprehensible and well-accepted tool to assess pain in PWH. Good convergent validity, internal consistency, reliability and reproducibility over 3 months were demonstrated for the intensity and interference dimensions of the questionnaire.

The multidimensionality of the MHPQ provides a complete characterization of pain, grasping a broad range of information. The questions focus on pain characteristics (locations, duration, frequency, temporal pattern, triggers, intensity and interference), as well as treatment options (pain specialists and management strategies) and satisfaction, which are not fully covered by other haemophilia questionnaires. For instance, data from this sample highlight that PWH may have multiple painful locations and experience acute and chronic pain simultaneously, underlining the pervasiveness of this problem and the need for a complete assessment. Additionally, only 48.8% of patients were satisfied with pain treatment, suggesting room for improvement in this field and supporting the relevance of a thorough assessment, to inform better care delivery. This is particularly relevant considering the high prevalence of pain in this sample, with 78.8% of the participants reporting pain due to haemophilia in the previous year. Similar figures were described in other investigations, with as much as 85% of respondents having had pain in a six-month period [16], or 81% of patients with severe haemophilia reporting pain [56]. On other studies, about half the participants dealt with daily arthritic joint pain [26,57]. Specifically concerning pain chronicity, recent surveys pointed to a prevalence ranging from 35 to 66% [10,16,58], but data were based on single questions rather than on a standardized definition of chronic pain, hindering comparisons across studies. To surpass this limitation, the MHPQ considers the definition for chronic pain in haemophilia proposed by the EHTSB [10]. Indeed, there is a need to clearly distinguish between acute and chronic pain, namely to adjust pain treatments to specific pain characteristics [10].

Regarding pain treatments, the participants selected a variety of management strategies, from pharmacotherapy to non-pharmacological approaches and complementary therapies, matching the recommendations stating that non-pharmacological treatments should be considered for haemophilia-related pain management [9,10]. Data from this study confirm that the RICE paradigm (rest, ice, compression and elevation) is frequently used among PWH, along with pain medication and clotting factor infusions. To investigate if each strategy is adequately adopted, it would be relevant to evaluate its use in acute vs. chronic pain situations. For example, clotting factor replacement should only be administered during active bleeding and not to control chronic pain, but it has been shown that patients have difficulty in distinguishing the two situations, sometimes using clotting factor replacement to treat persistent pain [13,18].

The analysis of item frequency showed that the highest percentage of missing responses concerned “pain duration”. This may be explained by the specificities of haemophilia pain, which can be present since childhood, thus explaining some difficulty in accurately determining pain duration. Nonetheless, we believe this item should remain in the questionnaire due to the recognized importance of this dimension for pain assessment. This issue could be settled by changing the item from an open-ended to a closed question with response options that included longer pain durations (*e*.*g*. from “a few days” to “since childhood”).

To analyse the factor structure of the pain intensity and pain interference dimensions, the items were included in EFA, which supported the underlying two-factor structure. Concerning the interference subscale, CFA further confirmed its adequacy to assess pain interference among PWH, by revealing adequate goodness-of-fit indices. Moreover, the reliability of both dimensions was demonstrated by appropriate internal consistency (Cronbach’s alpha) and test-retest reliability values.

Regarding the internal validity of pain intensity and pain interference dimensions, it is shown that the removal of any item decreases Cronbach’s alpha, asserting their relevance. The only exception is the “bleeding episodes” item (intensity dimension), which also had the lowest item-total correlation, and below-threshold inter-item correlations with three items in the subscale. This fact seemingly illustrates the distinct nature of acute and chronic pain in haemophilia, since the remaining items pertain to situations typically associated with chronic pain, while pain during bleeds is inherently acute. Despite its apparent poor performance, the item was kept in the subscale since pain intensity assessment in haemophilia cannot exclude haemarthrosis-related pain.

Pain intensity has been previously reported among PWH, though not focusing on specific situations but rather on least, worst and average scores [8,16,17,56], which might not adequately capture the reality of haemophilia-related pain. Other studies have assessed intensity for acute vs. persistent/chronic pain, but without discriminating intensity according to distinct triggers [13,14,18,59]. Therefore, rating pain intensity in distinct situations, in addition to a pain intensity global score, is a definite strength of this tool. This has increased utility for clinical practice, since more detailed data can be drawn to inform intervention, according to each specific situation.

Considering pain interference, inter-item correlations show strong associations (r>0.70) between three item-pairs, the highest between “general activity” and “normal work”. This was also evident in CFA, with the modification indices suggesting correlations between the errors of strongly related items to improve model’s fit. Interestingly, these results are in line with the two separate dimensions that have been proposed for the Brief Pain Inventory interference subscale, each translating more affective (mood, relations with people and enjoyment of life) or activity-related (general activity, walking ability, normal work and sleep) functions [60,61]. Indeed, it would be appealing to further explore the structure of this scale among PWH, since a distinction between affective/activity interference could provide relevant and complementary information. In the clinical setting, this could help shape more tailored interventions, with a particular focus either on reducing disability or promoting emotional well-being. In this sample, the highest interference scores were reported for “walking ability”, “normal work” and “general activity”, in line with recent studies, where most patients reported a negative impact of haemophilia on employment, daily, and recreational activities [8,19,26,62,63]. In fact, these results translate activity-related interference, in a close parallelism with the proposed BPI dimension previously discussed. On the other hand, the lowest mean interference score concerned a more affective and interpersonal dimension (“relations with other people”), as in other studies [8]. Actually, this is congruent with some investigations of quality-of-life among PWH, which report a more detrimental effect of haemophilia on physical, rather than on mental health domains [8,18,64,65].

Convergent validity of the MHPQ was shown by confirmation of most of the hypothesised correlations between MHPQ dimensions and clinical outcomes and validating measures, which underscores the utility of this questionnaire for clinical practice. Moreover, the MHPQ dimensions are still associated with some clinical outcomes assessed later, at the three month follow-up, namely the number of affected joints and pain frequency (weekly), therefore suggesting also a potential predictive value of the MHPQ.

Also meaningful is the association of the MHPQ dimensions with the pain subscale from the A36 Hemofilia-QoL, demonstrating that results provided by the MHPQ are congruent with other validated pain haemophilia-related measures. Convergent validity analysis also highlighted the association of pain with worse psychosocial and functional haemophilia-related outcomes, corroborating other findings regarding the negative impact of pain on emotional well-being [16,17,26], quality-of-life [16,19,57,66] and functionality [8,56], which further supports the utility of the MHPQ. Undoubtedly, these associations merit further exploration in future longitudinal studies, in order to better inform clinical intervention and the development of recommendations to improve patients’ care.

### Strengths and limitations

The MHPQ represents an important contribution to improve pain assessment among PWH and has clear strengths. It is a disease-specific measure, therefore contributing for a more thorough characterization of haemophilia pain than that provided by generic questionnaires. This initial validation shows the suitability of the questionnaire, encouraging its use and further validation in future studies. Data were obtained from a nationwide survey, rather than focusing on a single centre, considerably increasing findings generalizability. The inclusion of specific questions focusing on the distinction between acute and chronic pain contributes to differentiate this questionnaire from other haemophilia measures.

Nonetheless, some limitations of this study should be considered. Despite being adequate to assess pain across all ages, this paper only presents data from an adult sample, which is an important limitation. Further studies reporting data from children and teenagers are warranted, to confirm the suitability of the age-specific adaptations and to assess questionnaire validity for these age ranges. Also, the present sample size warrants caution when drawing conclusions regarding validity. However, it needs to be considered that haemophilia is a rare disease, with an estimated prevalence of only 700 cases in Portugal [45].

Another limitation worth considering is the fact that no other well-established pain measure was answered by the participants in this study, besides the MHPQ. Though this would have been useful to further demonstrate the validity and utility of the MHPQ, we opted not to add a general pain measure to this study. This decision was made to avoid patient burden, but also because we believe that existing pain measures do not adequately fit the specificities of haemophilia pain, particularly the possibility of having concomitant chronic and acute pain and multiple painful locations. As an example, ratings based on “worst”, “least” or “average” pain scores could be difficult to understand by patients, given the likelihood of having more than one pain location, which could leave doubts regarding what pain they were being asked about. In addition, these answers would also be strongly inflated if a bleeding episode had occurred in the previous 24 hours. Nonetheless, despite not having a global pain measure, the associations found between the MHPQ dimensions and the pain subscale from the A36 Hemofilia-QoL contribute to demonstrate the utility of the questionnaire to assess haemophilia-related pain.

Relevant research directions include examining the psychometric properties of the MHPQ with larger samples, comparing its responsiveness with other pain measures and assessing its cross-cultural validity. Longitudinal design studies are also warranted to establish more robust associations of the MHPQ with haemophilia outcomes (*e*.*g*. pain development over time, medication intake, need for orthopaedic surgery) and explore its sensibility to fluctuations in disease course or changes in treatment.

### Conclusion

The MHPQ is a promising tool to capture pain experience among PWH. It has unique features that focus on distinct pain dimensions, relevant to better understand haemophilia-related pain and to provide a comprehensive picture of each patients’ pain. In the clinical context, information provided by the questionnaire can help guide treatment, elicit further exploration of relevant issues and promote dialogue between patients and clinicians. Therefore, the MHPQ contributes to fill the gap concerning pain assessment in haemophilia and to improve pain management among this population. Ultimately, better knowledge of patients pain will contribute to more effective treatment approaches.

## Supporting information

S1 File Multidimensional Haemophilia Pain Questionnaire (MHPQ)–Portuguese version. (PDF)

S2 File Multidimensional Haemophilia Pain Questionnaire (MHPQ)–English version. (PDF)
