# Supplementary material for: A new measure to assess pain in people with haemophilia: The Multidimensional Haemophilia Pain Questionnaire (MHPQ)
Source: PLoS One. 2018 Nov 28;13(11):e0207939. doi: 10.1371/journal.pone.0207939 (PMC6261605; doi:10.1371/journal.pone.0207939)
Supplement: S1 File — (PDF) [file pone.0207939.s001.pdf]

QUESTIONÁRIO MULTIDIMENSIONAL DE DOR NA HEMOFILIA

Patrícia Ribeiro Pinto, Ana Cristina Paredes & Armando Almeida, 2018
Instituto de Investigação em Ciências da Vida e da Saúde, Escola de Medicina, Universidade do Minho

A dor é um dos principais sintomas associados à hemofilia, que pode ser muito incapacitante. Para melhorar os cuidados prestados, é importante para todos os profissionais de saúde compreender melhor esta dor, pelo que gostaríamos que nos falasse da sua experiência com a dor relacionada com a hemofilia.

1. **Alguma vez** sentiu dores relacionadas com a hemofilia? Não ❑ Sim ❑
2. Sentiu dores relacionadas com a hemofilia **no último ano**? Não ❑ Sim ❑

(Se respondeu NÃO, o questionário termina aqui)

3. A dor relacionada com a hemofilia apareceu **há mais de 3 meses**? Não ❑ Sim ❑
4. A dor relacionada com a hemofilia surge **mais do que uma vez por semana**? Não ❑ Sim ❑

Pensando nas dores que sentiu no **último ano**:

**Localização**	Assinale **todos** os locais onde **sente** ou **sentiu** dor no último ano	Assinale o local que lhe **dói** ou **doeu mais** no último ano (Assinale apenas 1 opção)	Assinale o local cuja dor **mais prejudicou** a sua vida no último ano (**teve maior impacto**) (Assinale apenas 1 opção)
Ombro direito	❑	❑	❑
Ombro esquerdo	❑	❑	❑
Cotovelo direito	❑	❑	❑
Cotovelo esquerdo	❑	❑	❑
Pulso direito	❑	❑	❑
Pulso esquerdo	❑	❑	❑
Anca direita	❑	❑	❑
Anca esquerda	❑	❑	❑
Joelho direito	❑	❑	❑
Joelho esquerdo	❑	❑	❑
Tornozelo direito	❑	❑	❑
Tornozelo esquerdo	❑	❑	❑
Outras localizações:			
________________	❑	❑	❑
________________	❑	❑	❑
________________	❑	❑	❑
________________	❑	❑	❑

No espaço abaixo, escreva (copie) o nome da **localização cuja dor mais prejudicou a sua vida no último ano e que assinalou na última coluna da tabela da página anterior** (Por exemplo, se na última coluna assinalou o espaço em frente à localização "joelho direito", em baixo deverá escrever também "joelho direito"):

Responda agora, por favor, às seguintes questões
PENSANDO APENAS NA DOR DA LOCALIZAÇÃO QUE ESCREVEU ACIMA

5. Aproximadamente, **há quanto tempo** apareceu essa dor? _________________

6. **Quando** costuma/costumava doer? (assinale todas as opções que se se aplicam)
   a) Quando me magoo ou tenho uma hemorragia ❑
   b) Quando faço esforços ou certos movimentos ❑
   c) Todas as semanas, mas tenho dias sem dor ❑
   d) Todos os dias, mas tenho momentos sem dor ❑
   e) A dor está sempre presente, é contínua e constante ❑
   f) Outro: ____ ❑

7. Quando foi a **última vez** que doeu?
   Hoje ❑ Na última semana ❑ No último mês ❑ Entre 1 e 6 meses ❑ Entre 6 e 12 meses ❑

8. Em que **momento do dia** dói mais?
   Manhã ❑ Durante a tarde ❑ Final do dia ❑ À noite ❑ Depende ❑

9. Quais as **razões ou motivos** que associa à dor? (assinale todas as opções que se se aplicam)
   a) A dor surge quando tenho uma hemorragia ❑
   b) A dor surge quando faço algum esforço e/ou durante o movimento, quando tenho que realizar tarefas (ex. pegar em pesos) ou andar ❑
   c) A dor surge quando subo ou desço escadas ❑
   d) A dor surge depois de descansar ou estar parado (ex. após estar muito tempo deitado, como de manhã ao acordar ou quando me levanto de uma cadeira) ❑
   e) A dor surge mesmo quando estou quieto, parado, sentado ou deitado (ex. à noite quando estou deitado na cama) ❑
   f) A dor surge se fizer algum movimento acidental (movimento em falso, "sem querer") ❑
   g) A dor surge quando há mudança das condições atmosféricas (sensibilidade às mudanças de tempo) ❑
   h) A dor está sempre presente, independentemente das circunstâncias ❑
   i) Outro motivo: ____ ❑

10. Para cada uma das situações descritas abaixo, assinale com um círculo, na escala de 0 a 10, o número que melhor descreve a **intensidade de dor habitual** que costuma sentir **na localização que assinalou anteriormente**, sendo que o valor <u>“zero” corresponde a ausência de dor e “dez” corresponde à pior dor que pode imaginar.</u>

a. Episódios de hemorragia

0	1	2	3	4	5	6	7	8	9	10
Sem dor	Dor ligeira			Dor moderada			Dor severa			A pior dor que pode imaginar

b. Quando faço algum esforço e/ou durante o movimento, quando tenho que realizar tarefas (ex. pegar em pesos) ou andar

0	1	2	3	4	5	6	7	8	9	10
Sem dor	Dor ligeira			Dor moderada			Dor severa			A pior dor que pode imaginar

c. Quando subo ou desço escadas

0	1	2	3	4	5	6	7	8	9	10
Sem dor	Dor ligeira			Dor moderada			Dor severa			A pior dor que pode imaginar

d. Depois de descansar ou estar parado (ex. após estar muito tempo deitado, como de manhã ao acordar ou quando me levanto de uma cadeira)

0	1	2	3	4	5	6	7	8	9	10
Sem dor	Dor ligeira			Dor moderada			Dor severa			A pior dor que pode imaginar

e. Quanto estou quieto, parado, sentado ou deitado (ex. à noite quando estou deitado na cama)

0	1	2	3	4	5	6	7	8	9	10
Sem dor	Dor ligeira			Dor moderada			Dor severa			A pior dor que pode imaginar

f. Quando faço algum movimento acidental (movimento em falso, “sem querer”)

0	1	2	3	4	5	6	7	8	9	10
Sem dor	Dor ligeira			Dor moderada			Dor severa			A pior dor que pode imaginar

11. Para cada uma das situações descritas abaixo, assinale com um círculo, na escala de 0 a 10, o número que melhor descreve a **interferência que a dor da articulação que assinalou anteriormente** costuma ter em cada uma das seguintes situações, sendo que <u>o valor “zero” corresponde a nenhuma interferência e “dez” corresponde à máxima interferência.</u>

a. Atividade Geral (todas as atividades do dia-a-dia)

0	1	2	3	4	5	6	7	8	9	10
Sem interferência	Interfere ligeiramente			Interfere moderadamente			Interfere severamente			Interfere completamente

b. Disposição (humor, estado de ânimo)

0	1	2	3	4	5	6	7	8	9	10
Sem interferência	Interfere ligeiramente			Interfere moderadamente			Interfere severamente			Interfere completamente

c. Capacidade para andar a pé

0	1	2	3	4	5	6	7	8	9	10
Sem interferência	Interfere ligeiramente			Interfere moderadamente			Interfere severamente			Interfere completamente

d. Trabalho normal (inclui tanto o trabalho doméstico como o trabalho fora de casa)

0	1	2	3	4	5	6	7	8	9	10
Sem interferência	Interfere ligeiramente			Interfere moderadamente			Interfere severamente			Interfere completamente

e. Relações com outras pessoas (relacionamento com amigos, familiares ou colegas)

0	1	2	3	4	5	6	7	8	9	10
Sem interferência	Interfere ligeiramente			Interfere moderadamente			Interfere severamente			Interfere completamente

f. Sono

0	1	2	3	4	5	6	7	8	9	10
Sem interferência	Interfere ligeiramente			Interfere moderadamente			Interfere severamente			Interfere completamente

g. Prazer de viver

0	1	2	3	4	5	6	7	8	9	10
Sem interferência	Interfere ligeiramente			Interfere moderadamente			Interfere severamente			Interfere completamente

12. A tabela seguinte apresenta algumas **estratégias que as pessoas usam para aliviar a dor**. Assinale as estratégias que utiliza habitualmente, ou que já utilizou, e indique, numa **escala de 0% a 100%**, a percentagem de alívio que sente ou sentiu com cada uma delas.

	Faço ou já fiz isto	Percentagem de alívio (0% - 100%)
Medicação para as dores (analgésicos)/Pomadas*	❑	______ %
Gelo	❑	______ %
Descanso/Repouso	❑	______ %
Rezar/Orar	❑	______ %
Elevar a zona dolorosa/Mudar de posição	❑	______ %
Administração de fator	❑	______ %
Relaxamento	❑	______ %
Aplicação de calor	❑	______ %
Compressão	❑	______ %
Bebidas alcoólicas	❑	______ %
Fumar tabaco	❑	______ %
Drogas recreativas (ex. cannabis)	❑	______ %
Massagens terapêuticas	❑	______ %
Reiki/Meditação	❑	______ %
Produtos naturais/Homeopatia/Naturopatia	❑	______ %
Técnicas de distração (ex. ir fazer outra coisa que gosto)	❑	______ %
Procura de apoio/companhia de familiares e/ou amigos	❑	______ %

Outras estratégias para lidar com a dor:		
___________________________________	❑	______ %
___________________________________	❑	______ %
___________________________________	❑	______ %

* Se assinalou esta opção, indique o nome da medicação para as dores (analgésicos) e/ou pomadas que costuma usar:

____

____

13. Assinale, na lista de especialidades / terapias assinaladas abaixo, as **que já consultou** (ou **gostaria de consultar)** para ajudar a lidar com a dor:

	Já consultei	Gostaria de consultar
Médicos da Hemofilia (Imunohemoterapeutas)	❑	❑
Médico de família	❑	❑
Ortopedista	❑	❑
Anestesiologista	❑	❑
Fisiatra	❑	❑
Psiquiatra	❑	❑
Psicólogo	❑	❑
Fisioterapeuta	❑	❑
Acupuntura	❑	❑
Meditação	❑	❑
Reiki	❑	❑
Outro:		
____	❑	❑
____	❑	❑

OU Não consultei nenhuma destas especialidades / terapias para ajudar a lidar melhor com a dor ❑

13. Globalmente, qual é o seu grau de **satisfação geral** em relação à forma como a sua dor tem sido tratada pelos profissionais de saúde?

Muito insatisfeito	Insatisfeito	Nem insatisfeito nem satisfeito	Satisfeito	Muito Satisfeito
❑	❑	❑	❑	❑

VERIFIQUE POR FAVOR SE RESPONDEU A TODAS AS QUESTÕES.
Muito obrigado pela sua colaboração.
