# Supplementary material for: A new measure to assess pain in people with haemophilia: The Multidimensional Haemophilia Pain Questionnaire (MHPQ)
Source: PLoS One. 2018 Nov 28;13(11):e0207939. doi: 10.1371/journal.pone.0207939 (PMC6261605; doi:10.1371/journal.pone.0207939)
Supplement: S2 File — (PDF) [file pone.0207939.s002.pdf]

MULTIDIMENSIONAL HAEMOPHILIA PAIN QUESTIONAIRE

Experimental research version - English translation not yet validated.

Patrícia Ribeiro Pinto, Ana Cristina Paredes & Armando Almeida, 2018
Life and Health Sciences Research Institute, School of Medicine, University of Minho

Pain is one of the main symptoms associated with haemophilia, which can cause great disability. To improve the quality of care, it is important for every healthcare professional to better understand this pain. Therefore, we would like you to tell us about your experience with haemophilia-related pain.

1. **Have you ever** had pain due to haemophilia? No ❑ Yes ❑
2. Have you had pain due to haemophilia **during the past year?** No ❑ Yes ❑

(If the answer is NO, the questionnaire ends here)

3. Has the pain related to haemophilia **started over 3 months ago**? No ❑ Yes ❑
4. Does the pain related to haemophilia **occur more than once a week?** No ❑ Yes ❑

Thinking about the pain you had during the **past year**:

**Location**	Mark **all** the locations where you **have** or **had** pain during the past year	Mark the location that **is** or **was the most painful** during the past year (Choose 1 option only)	Mark the location in which pain had the **most negative impact** on your life during the past year (Choose 1 option only)
Right shoulder	❑	❑	❑
Left shoulder	❑	❑	❑
Right elbow	❑	❑	❑
Left elbow	❑	❑	❑
Right wrist	❑	❑	❑
Left wrist	❑	❑	❑
Right hip	❑	❑	❑
Left hip	❑	❑	❑
Right knee	❑	❑	❑
Left knee	❑	❑	❑
Right ankle	❑	❑	❑
Left ankle	❑	❑	❑
Other locations:			
________________	❑	❑	❑
________________	❑	❑	❑
________________	❑	❑	❑
________________	❑	❑	❑

In the space provided below, write (copy) the name of the **location in which pain had the most negative impact in the past year and that you marked in the last column of the table in the previous page** (For example, if in the last column you marked the space ahead of the location "right knee", you should also write "right knee" in the space bellow):

Now, please, answer the following questions
CONSIDERING EXCLUSIVELY THE <u>PAIN IN THE LOCATION YOU WROTE ABOVE</u>

5. Approximately, **how long ago** did that pain start? _________________

6. Usually, **when** does/did it hurt? (mark all that apply)
   a) After getting hurt or during bleeds ❑
   b) During physical efforts and/or movement ❑
   c) Every week, but I have days without pain ❑
   d) Every day, but I have some moments without pain ❑
   e) The pain is always present, it is continuous and constant ❑
   f) Other: ____ ❑

7. When was the **last time** it hurt?
   Today ❑ Last week ❑ Last month ❑ Between 1 and 6 months ❑ Between 6 and 12 months ❑

8. In what **moment of the day** is the pain more intense?
   Morning ❑ During the afternoon ❑ End of the day ❑ At night ❑ Depends ❑

9. Which **reasons or triggering factors** do you associate with pain? (mark all that apply)
   a) Pain occurs during bleeding episodes ❑
   b) Pain occurs during physical efforts and/or movement, when I do certain tasks (like picking up heavy weights) or while walking ❑
   c) Pain occurs when climbing up or down stairs ❑
   d) Pain occurs after resting or staying still (e.g. after lying down for a long time, like in the morning after waking up or when getting up from a chair) ❑
   e) Pain occurs even during rest, staying still, sitting or lying down (e.g. at night when I am lying in bed) ❑
   f) Pain occurs due to accidental or "wrong" movements ❑
   g) Pain occurs due to weather changes ❑
   h) Pain is always present, no matter the circumstances ❑
   i) Other motive: ____ ❑

10. For each of the situations described below, mark with a circle, according to the 0-10 scale, the number that best describes the **pain intensity** you usually have in the location previously selected. Note that the number “zero” represents absence of pain and “ten” represents the worst pain imaginable.

a. Bleeding episodes

0	1	2	3	4	5	6	7	8	9	10
No pain	Mild pain			Moderate pain			Severe pain			Worst possible pain

b. During physical efforts and/or movement, when I do certain tasks (like picking up heavy weights) or while walking

0	1	2	3	4	5	6	7	8	9	10
No pain	Mild pain			Moderate pain			Severe pain			Worst possible pain

c. When climbing up or down stairs

0	1	2	3	4	5	6	7	8	9	10
No pain	Mild pain			Moderate pain			Severe pain			Worst possible pain

d. After resting or staying still (e.g. after lying down for a long time, like in the morning after waking up or when getting up from a chair)

0	1	2	3	4	5	6	7	8	9	10
No pain	Mild pain			Moderate pain			Severe pain			Worst possible pain

e. During rest, staying still, sitting or lying down (e.g. at night when I am lying in bed)

0	1	2	3	4	5	6	7	8	9	10
No pain	Mild pain			Moderate pain			Severe pain			Worst possible pain

f. After accidental or “wrong” movements

0	1	2	3	4	5	6	7	8	9	10
No pain	Mild pain			Moderate pain			Severe pain			Worst possible pain

11. For each of the situations described below, mark with a circle, according to the 0-10 scale, the number that best describes **pain interference in the location previously selected**. Note that the number “zero” represents no interference and “ten” represents maximum interference.

a. General activity

0	1	2	3	4	5	6	7	8	9	10
Does not interfere	Mild interference			Moderate interference			Severe interference			Completely interferes

b. Mood

0	1	2	3	4	5	6	7	8	9	10
Does not interfere	Mild interference			Moderate interference			Severe interference			Completely interferes

c. Walking ability

0	1	2	3	4	5	6	7	8	9	10
Does not interfere	Mild interference			Moderate interference			Severe interference			Completely interferes

d. Normal work (includes both work outside the home and housework)

0	1	2	3	4	5	6	7	8	9	10
Does not interfere	Mild interference			Moderate interference			Severe interference			Completely interferes

e. Relations with other people

0	1	2	3	4	5	6	7	8	9	10
Does not interfere	Mild interference			Moderate interference			Severe interference			Completely interferes

f. Sleep

0	1	2	3	4	5	6	7	8	9	10
Does not interfere	Mild interference			Moderate interference			Severe interference			Completely interferes

g. Enjoyment of life

0	1	2	3	4	5	6	7	8	9	10
Does not interfere	Mild interference			Moderate interference			Severe interference			Completely interferes

12. The following table presents some **strategies people use to relieve their pain**. Mark the strategies you usually use, or ever used, and write, on a **0% to 100% scale**, how much relieve does each strategy provide (or has provided).

	I do or did this	Percentage of relief (0% - 100%)
Pain medication (analgesics)/Ointments*	❑	______ %
Ice	❑	______ %
Rest	❑	______ %
Praying	❑	______ %
Elevation of the painful location/Changing position	❑	______ %
Clotting factor replacement	❑	______ %
Relaxing techniques	❑	______ %
Heat	❑	______ %
Compression	❑	______ %
Alcohol	❑	______ %
Tobacco	❑	______ %
Recreational drugs (e.g. cannabis)	❑	______ %
Therapeutic massage	❑	______ %
Reiki/Meditation	❑	______ %
Natural products/Homeopathy/Naturopathy	❑	______ %
Distracting techniques (e.g. do other things you enjoy)	❑	______ %
Search for support/company of family and/or friends	❑	______ %

Other strategies to deal with pain:		
________________________________	❑	______ %
________________________________	❑	______ %
________________________________	❑	______ %

* If you marked this option, write the name of pain medication (analgesics) and/or ointments you usually use:____
____

13. Mark, on the following list of specialties /therapies, the ones you **have consulted** (or **wish to consult**) to help dealing with pain:

	Have consulted	Wish to consult
Haemophilia doctor	❑	❑
Family doctor	❑	❑
Orthopedist	❑	❑
Anesthesiologist	❑	❑
Physiatrist	❑	❑
Psychiatrist	❑	❑
Psychologist	❑	❑
Physical therapy/Physiotherapist	❑	❑
Acupuncture	❑	❑
Meditation	❑	❑
Reiki	❑	❑
Other:		
____	❑	❑
____	❑	❑

OR I have not consulted any specialty / therapy to help dealing with pain ❑

13.Globally, what is you **satisfaction level** with current pain treatment by healthcare professionals?

Very dissatisfied	Dissatisfied	Neither satisfied nor dissatisfied	Satisfied	Very satisfied
❑	❑	❑	❑	❑

PLEASE MAKE SURE YOU ANSWERED ALL THE QUESTIONS.
Thank you very much for your collaboration.
